# Unveiling the Gaps: Machine Learning Models for Unmeasured Ions

**DOI:** 10.3390/diagnostics16030427

**Published:** 2026-02-01

**Authors:** Furkan Tontu, Zafer Çukurova

**Affiliations:** 1 Department of Anesthesiology and Reanimation, Başakşehir Çam and Sakura City Hospital, Istanbul 34480, Turkey; 2 Department of Reanimation, Bakirkoy Dr. Sadi Konuk Training and Research Hospital, Istanbul 34147, Turkey; zcukurova@gmail.com

**Keywords:** unmeasured ions, acid–base balance, machine learning, arterial blood gas, anion gap, strong ion gap, base excess gap, intensive care, XGBoost

## Abstract

**Background:** Unmeasured ions (UIs) contribute significantly to acid–base disturbances in critically ill patients, yet the optimal parameter for their estimation remains uncertain. The most widely used indicators are the albumin-corrected anion gap (AGc), the strong ion gap (SIG), and the base excess gap (BEGap). **Methods:** In this retrospective cohort study, a total of 2274 ICU patients (2018–2022) were included in the development cohort, and an independent external validation cohort of 1202 patients (2023–2025) was used to assess temporal generalizability. Three approaches to blood gas analysis—traditional (PaCO_2_, HCO_3_^−^, AGc), Stewart (PaCO_2_, SIDa, ATOT, SIG), and partitioned base excess (PaCO_2_, BECl, BEAlb, BELac, BEGap)—were evaluated. Multivariable linear regression (MLR) and machine learning (ML, random forest [RF], extreme gradient boosting [XGBoost], and support vector regression [SVR]) were applied to evaluate the explanatory performance of analytical approaches with respect to arterial pH. Model performance was assessed using adjusted R^2^, RMSE, and MAE. Variable importance was quantified with tree-based methods, SHAP values, and permutation importance. All modeling and reporting steps followed the PROBAST-AI guideline. **Results:** In multiple linear regression (MLR), the partitioned base excess (BE) approach achieved the highest explanatory performance (adjusted R^2^ = 0.949), followed by the traditional (0.929) and Stewart approaches (0.926). In ML analyses, model fit was high across all approaches. For the traditional approach, R^2^ values were 0.979 with RF, 0.974 with XGBoost, and 0.934 with SVR. The Stewart’s approach showed lower overall explanatory performance, with R^2^ values of 0.876 (RF), 0.967 (XGBoost), and 0.996 (SVR). The partitioned BE approach again demonstrated the strongest explanatory performance, achieving R^2^ values of 0.975 with XGBoost and 0.989 with SVR. Across all analytical models, BEGap consistently emerged as a strong and independent determinant of arterial pH, outperforming SIG and AGc. SIG showed an intermediate contribution, whereas AGc provided minimal independent explanatory value. Among ML models, XGBoost showed the most stable and accurate explanatory performance across approaches. **Conclusions:** This study demonstrates that BEGap is a practical, physiologically informative, and bedside-applicable parameter for assessing unmeasured ions, outperforming both AGc and SIG across linear and non-linear analytical models.

## 1. Introduction

Acid–base disturbances are associated with morbidity and mortality in critically ill patients [1]. Unmeasured ions (UIs) such as ketoacids, Krebs cycle intermediates (citric acid, acetate, fumarate), sulfate, urate, hydroxypropionate, oxalate, furanpropionate, and those accumulating after drug intoxication may significantly influence acid–base balance, yet they are not routinely measured [2,3]. Therefore, clinicians have adopted different parameters to identify these UIs.

In the traditional approach (Henderson–Hasselbalch), pH is primarily determined by partial pressure of carbon dioxide (PaCO_2_) and bicarbonate (HCO_3_). Yet these parameters are insufficient to explain the contribution of UIs [4]. To address this, clinicians have employed different parameters [5]. The most common is the anion gap (AG), a practical tool in the differential diagnosis of metabolic acidosis [6,7]. However, AG often requires correction for albumin (AGc), and its dependence on phosphate and particularly lactate levels limits its clinical utility [8,9,10].

In the Stewart approach, the primary determinants of pH are the strong ion difference (SID), total weak acids (A_TOT_), and PaCO_2_ [11]. The strong ion gap (SIG), a component of this approach, reveals the presence of UIs more specifically than AGc [11,12]. However, its complex calculation and limited feasibility in routine clinical practice remain major drawbacks [13,14].

The partitioned base excess (BE) approach, which separates standard base excess (SBE) into four components, has recently emerged and gained increasing use [15,16,17,18]. In this approach, the effects of sodium-chloride (BECl), lactate (BELac), and albumin (BEAlb) on SBE are calculated, and the remaining component is assumed to represent UIs (BEGap) [15,16]. The main advantage of this method is its straightforward calculation and high feasibility at the bedside [6,15,16].

No gold standard parameter has been established to represent UIs, and clinicians have therefore sought to determine which parameter reflects them most accurately [5]. The three main parameters—AGc, SIG, and BEGap—have generally been studied in relation to morbidity and mortality across different patient groups, with conflicting results [19,20,21,22]. The effects of these parameters on pH, however, have not been sufficiently investigated [23].

In recent years, machine learning (ML) and artificial intelligence (AI) have been increasingly employed in clinical medicine for diagnostic classification, risk prediction, and decision support [24,25]. ML models have been used to determine COVID-19 diagnosis and prognosis based on blood gas parameters, and have been shown to predict the need for ICU [26]. In a large cohort, ML algorithms were reported to classify acid–base disorders into 15 distinct categories with accuracy rates exceeding 99% [27]. ML may serve as a useful analytical approach for characterizing the status of critically ill patients and guiding the management of acid–base and potassium imbalances [28]. ML models have also been applied to ensure data integrity in ICU settings, accurately distinguishing arterial from venous samples with performance rates above 99% [29]. Furthermore, recent comprehensive reviews have highlighted the potential of ML and deep learning approaches to classify acid–base disturbances, predict disease severity, and support clinical decision-making using ABG data [30].

The primary aim of the present study was to evaluate the associations between AGc, SIG, and BEGap and arterial pH, and to determine which parameter most reliably reflects UIs from a physiological perspective. The secondary aim was to compare how three analytical approaches—traditional, Stewart, and partitioned BE—account for variability in arterial pH and characterize unmeasured ions within the context of acid–base physiology.

Accordingly, this study was designed to address an important gap in the literature by providing a comprehensive analytical comparison of unmeasured ion approaches. To achieve this, the study was conducted with following specific objectives:

1. To examine the independent associations of AGc, SIG, and BEGap with arterial pH in a large cohort of critically ill patients.
2. To benchmark traditional, Stewart, and partitioned BE approaches using both multiple linear regression (MLR) and machine learning (ML) models under linear and non-linear modeling assumptions.
3. To evaluate the robustness and temporal external generalizability of these analytical approaches using an independent validation cohort.
4. To explore potential bedside clinical implications through representative clinical cases, illustrating how BEGap-based interpretation may complement conventional approaches.

## 2. Methods

### 2.1. Study Design and Population

This retrospective, cross-sectional cohort study was conducted in the intensive care unit (ICU) of the University of Health Sciences, Bakirkoy Dr. Sadi Konuk Training and Research Hospital between January 2018 and June 2025. A total of 8468 consecutive adult patients (≥18 years) admitted during this period were screened for eligibility.

The study protocol was reviewed and approved by the Ethics Committee of Bakirkoy Dr. Sadi Konuk Training and Research Hospital on 20 August 2025 (Approval No: 2025/260, Decision No: 2025-15-14).

After applying exclusion criteria, 3476 patients with complete paired arterial blood gas (ABG) and serum biochemistry results at ICU admission were included in the analysis. Arterial blood gas analyses were performed using a Radiometer ABL 800 FLEX (Radiometer Medical ApS, Copenhagen, Denmark).

To ensure temporal independence between model development and evaluation, the dataset was chronologically divided into two cohorts:

- The internal development cohort consisted of 2274 patients admitted between January 2018 and December 2022, used for model training and internal validation.
- The external validation cohort comprised 1202 patients admitted between January 2023 and June 2025, used exclusively for external performance evaluation.

This design allowed assessment of the model’s generalizability on future, unseen data. All modeling and reporting steps adhered to the PROBAST-AI recommendations [31] (see Table S1).

### 2.2. Missing Data Management

Patients were included if they had a valid arterial blood gas measurement and concurrent serum levels of albumin, magnesium, and phosphate (PO_4_) obtained at ICU admission. Patients were excluded if they had missing data in any required biochemical or blood gas parameter, were under 18 years of age, or had repeated ICU admissions.

**Figure 1:**
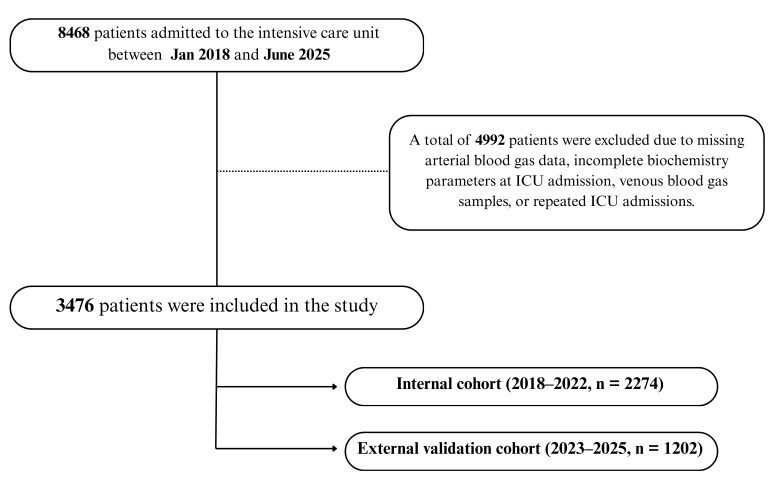
Patient flow diagram for model development and validation. A total of 8468 ICU admissions were screened; after exclusions, 3476 unique patients were included and chronologically divided into an internal development cohort (2018–2022, *n* = 2274) and an external validation cohort (2023–2025, *n* = 1202).

Of the 8468 patients screened, 4992 were excluded due to missing or clotted blood samples, incomplete biochemistry data, or venous instead of arterial gas measurements. This resulted in a final dataset of 3476 unique patients with complete-case data. Among these, 2274 belonged to the internal cohort (2018–2022) and 1202 to the external validation cohort (2023–2025), as illustrated in Figure 1.

All patients with incomplete or missing values were excluded prior to analysis, and no imputation procedures were applied. Therefore, all regression and ML models were trained and tested using a complete-case dataset, ensuring consistency and minimizing potential bias due to missing data handling.

### 2.3. Model Development and Validation

This study used ML models as complementary analytical tools to quantify how physiologically informative different acid–base frameworks are in characterizing arterial pH variability. The intention was not to develop a deployable diagnostic tool, but to benchmark analytical performance across linear and non-linear modeling assumptions. ML models complemented, rather than replaced, classical regression-based analysis by assessing non-linear behavior, calibration characteristics, and temporal external generalizability.

The internal dataset (2018–2022) was randomly divided into training (80%) and testing (20%) subsets for model development and performance assessment using a fixed random seed. Model hyperparameters were predefined and kept fixed across analyses based on commonly accepted configurations. Internal model stability was assessed using 10-fold cross-validation on the training set.

Four algorithms were evaluated: multiple linear regression (MLR), random forest (RF), extreme gradient boosting (XGBoost), and support vector regression (SVR). Model performance was reported using the coefficient of determination (R^2^), root mean square error (RMSE), and mean absolute error (MAE).

The contribution of framework-specific metabolic variables was quantified through ablation experiments, in which AGc, SIG, and BEGap were systematically removed from the traditional, Stewart, and partitioned BE feature sets, respectively. Predictive performance of the reduced models was compared with that of the corresponding full models using 10-fold paired cross-validation.

Calibration metrics—including calibration-in-the-large, intercept, and slope—were derived from test set predictions. For external validation, all final models were evaluated on an independent temporal cohort (2023–2025, *n* = 1202) to assess generalizability using the same performance and calibration metrics.

**Data Leakage Prevention**

Temporal separation between the internal (2018–2022) and external (2023–2025) cohorts was used for validation consistency, while specific measures to prevent data leakage are detailed below. To minimize the risk of data leakage, all preprocessing and model development steps—including normalization, feature scaling, and hyperparameter optimization—were performed within each training fold during cross-validation. No information from the validation folds was accessed during training, and a fixed random seed (42) was applied to ensure reproducibility. This procedure ensured that model performance reflected genuine generalization ability rather than contamination across data partitions.

**Collected variables**

Demographic characteristics (age, sex, BMI), clinical severity scores (APACHE II and SOFA), and outcomes (ICU mortality) were recorded. Laboratory variables included pH, partial pressure of carbon dioxide (PaCO_2_), bicarbonate (HCO_3_^−^), SBE, sodium (Na^+^), potassium (K^+^), chloride (Cl^−^), calcium (Ca^2+^), magnesium (Mg^2+^), lactate, albumin, and inorganic phosphorus (PO_4_).

**Calculated parameters**

The following formulas were used:

**Traditional Approach**

AG and AGc were calculated as follows [4]:

**AG** = (Na + K) − (Cl + HCO_3_)

**AGc** = AG + 0.25 × (42 − Albumin [g/L])

**Stewart’s Approach**

After converting Mg and PO_4_ to mmol/L, the parameters SIDa, SIDe, A_TOT_, and SIG were calculated in mmol/L using the following equations [11]:

**Mg (mmol/L)** = Mg (mg/dL) × 0.41152

**Pi (mmol/L)** = PO_4_ (mg/dL) × 0.323

**SIDa** = (Na + K + Ca + Mg) − (Cl + Lactate)

**SIDe** = (0.0301 × PaCO_2_ × 10^(pH−6.1)^) + [Alb]^−^ + [Pi]^−^

**[Alb^−^]** = (0.123 × pH − 0.631) × Albumin (g/L)

[**Pi^−^]** = Pi (mmol/L) × (0.309 × pH − 0.469)

**A_TOT_** = [Alb]^−^ + [Pi]^−^

**SIG** = SIDa − SIDe

**SIG** = [(Na + K + Ca + Mg) − (Cl + Lactate)] − [(0.0301 × PaCO_2_ × 10^(pH−6.1)^) + (0.123 × pH − 0.631) × Albumin (g/L) + Pi (mmol/L) × (0.309 × pH − 0.469)]

**Partitioned BE Approach**

After calculating BECl, BEAlb, and BELac, their sum was subtracted from SBE to obtain BEGap [15,16]:

**BECl** = Na − Cl − 35

**BEAlb** = 0.25 × (42 − Albumin [g/L])

**BELac** = 1 − Lactate

**BEGap** = SBE − BECl − BEAlb − BELac

All units are expressed in mmol/L unless otherwise specified.

#### 2.3.1. Statistical Analysis

Descriptive statistics were expressed as median with interquartile range (IQR, 25th–75th percentile) for continuous variables and as number with percentage (*n*, %) for categorical variables. Normality of distribution was assessed using the Kolmogorov–Smirnov test.

Three different approaches were compared:

1. Traditional approach (PaCO_2_, HCO_3_, AGc)
2. Stewart’s approach (PaCO_2_, SIDa, A_TOT_, SIG)
3. Partitioned base excess approach (PaCO_2_, BECl, BEAlb, BELac, BEGap)

#### 2.3.2. Regression Models

Multiple linear regression was first applied to quantify the contribution of each parameter to arterial pH. Model performance was evaluated using adjusted R^2^, Durbin–Watson statistics, and ANOVA F-test significance.

#### 2.3.3. Machine Learning Models

Three ML algorithms were implemented as complementary analytical tools to evaluate possible non-linear relationships and assess model fit:

- Random Forest (RF)
- Extreme Gradient Boosting (XGBoost)
- Support Vector Regression (SVR, RBF kernel)

Model hyperparameters were optimized using 10-fold cross-validation. Performance was quantified with R^2^, RMSE and MAE.

To assess internal validity and model calibration in accordance PROBAST-AI guideline, we additionally calculated [31]:

- Calibration-in-the-large (mean observed − predicted pH),
- Calibration slope and intercept,
- Brier-style mean squared error for continuous outcomes.

Model hyperparameters and preprocessing steps are reported in detail in the Supplementary Material Table S2.

#### 2.3.4. Feature Importance and Explainability

Variable importance was evaluated by complementary methods:

- Tree-based importance (TreeImp)—reduction in prediction error per variable in RF/XGBoost.
- SHAP values—game-theoretic contribution of each predictor.
- Permutation importance—change in prediction error after random shuffling (for SVR). A composite ranking was derived by averaging normalized TreeImp and SHAP scores.

All analyses were performed in Python 3.12 using scikit-learn (1.3), XGBoost (2.0), and SHAP (0.45). Two-sided *p* < 0.05 was considered statistically significant.

#### 2.3.5. Illustrative Clinical Cases

Three representative ICU cases were selected from the cohort to illustrate the potential clinical implications of the three approaches. For each case, acid–base status was interpreted using the Henderson–Hasselbalch, Stewart’s, and partitioned BE approaches, and relevant analytical parameters (AGc, SIDa, A_TOT_, SIG, BECl, BEAlb, BELac, and BEGap) were calculated as described above.

## 3. Results

### 3.1. Patient Characteristics

A total of 2274 patients were included in the development (2018–2022) cohort, with a median age of 68 years (IQR 57–79) and 1,383 males (61%). The median BMI was 26.4 kg/m^2^ (23.8–29.8). The median APACHE II and SOFA scores were 26 (22–31) and 7 (5–10), respectively, and overall mortality was 40%. The independent external validation cohort included 1202 patients admitted between 2023 and 2025. Baseline demographic and biochemical characteristics were broadly comparable to the development cohort. Patients in the external cohort were slightly younger, had a lower proportion of males, and exhibited modestly lower APACHE II scores and mortality rates (Table 1).

The distribution of admission diagnoses was as follows: renal disorders in 74 patients (3.3%), sepsis in 304 (13.4%), metabolic disorders in 59 (2.6%), circulatory disorders in 549 (24.1%), trauma in 181 (8.0%), intoxication in 57 (2.5%), gastrointestinal disorders in 72 (3.2%), postoperative cases in 147 (6.5%), respiratory disorders in 558 (24.5%), neurological conditions in 96 (4.2%), malignancy in 131 (5.8%), and hematologic disorders in 46 (2.0%) (Table S3). The distribution of ICU admission diagnoses for patients included in the external validation cohort (2023–2025) is also presented in Table S3.

Baseline arterial blood gas and biochemical parameters, including pH, electrolytes, albumin, lactate, and partitioned base excess values, are summarized in Table 1.

### 3.2. Multivariable Linear Regression Analysis

Using multiple linear regression, the traditional model (PaCO_2_, SBE, HCO_3_, AGc) achieved an adjusted R^2^ of 0.929 (Durbin–Watson: 2.11; *p* < 0.001). The Stewart model (PaCO_2_, SIDa, ATOT, SIG) demonstrated a slightly lower fit with an adjusted R^2^ of 0.926 (Durbin–Watson: 2.02; *p* < 0.001). The partitioned base excess model (BECl, BELac, BEAlb, BEGap, PaCO_2_) reached the highest explanatory performance (adjusted R^2^ = 0.949, Durbin–Watson: 1.89; *p* < 0.001) (see Table 2).

### 3.3. Machine Learning Performance

In ML models, RF using the traditional variables reached an R^2^ of 0.979, RMSE 0.015, and MAE 0.009. XGB showed a similar level of model fit with an R^2^ of 0.974, RMSE 0.017, and MAE 0.01, while SVR demonstrated lower model fit (R^2^ 0.934, RMSE 0.028, MAE 0.019). For the Stewart model, RF yielded an R^2^ of 0.876 (RMSE 0.038, MAE 0.023), XGB achieved 0.967 (RMSE 0.019, MAE 0.011), and SVR provided the highest model fit within this framework with 0.996 (RMSE 0.006, MAE 0.005). For the partitioned BE model, RF reached an R^2^ of 0.922 (RMSE 0.03, MAE 0.019), XGB achieved 0.975 (RMSE 0.017, MAE 0.011), and SVR again provided the highest model fit with 0.989 (RMSE 0.011, MAE 0.005) (see Table 3 and Table S4).

### 3.4. Internal Cross-Validation Performance

In the 10-fold cross-validation analysis, XGB yielded the highest model fit across all three blood gas models, with a mean ± SD R^2^ of 0.975 ± 0.010, RMSE of 0.017 ± 0.003, and MAE of 0.009 ± 0.001. RF (R^2^ 0.971 ± 0.014) and linear regression (R^2^ 0.971 ± 0.003) demonstrated comparable but slightly lower model fit, whereas SVR showed lower model fit and higher variability (R^2^ 0.930 ± 0.037, RMSE 0.029 ± 0.007) (See SDM Table S5).

#### 3.4.1. Calibration and Discrimination

**Figure 2:**
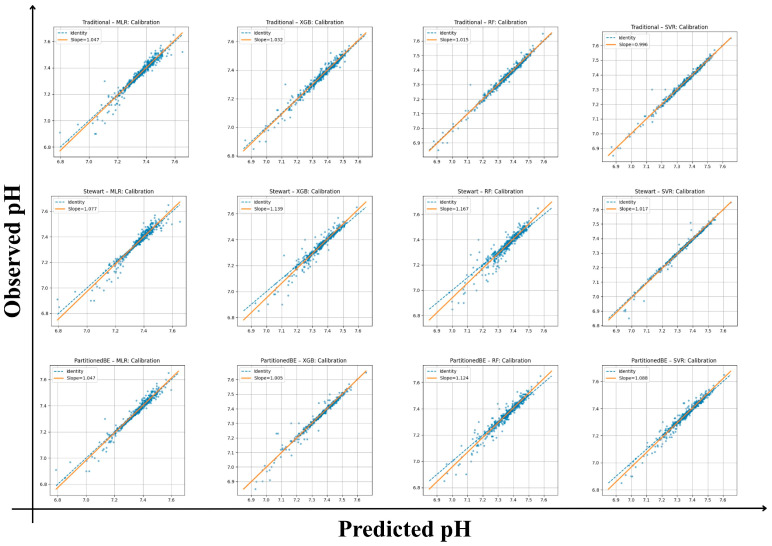
Calibration plots of predicted versus observed arterial pH across frameworks and algorithms. Each panel shows calibration of traditional, Stewart, and partitioned base excess (BE) models using multiple linear regression (MLR), random forest (RF), extreme gradient boosting (XGBoost), and support vector regression (SVR). Orange lines denote regression fit (slope/intercept); dashed lines show the identity line (perfect calibration).

All frameworks demonstrated strong internal calibration and model fit for arterial pH estimation (Table 3). Calibration slopes ranged from 0.99 to 1.17, with intercepts between −1.23 and +0.03, indicating good agreement between predicted and observed values across all models. Mean squared error (Brier-like) values were consistently low (0.00017–0.0015), supporting the reliability of predictions. Among the traditional framework models, the SVR achieved the highest internal model fit (R^2^ = 0.99) with the lowest RMSE (0.013) and MAE (0.008), reflecting excellent internal fit. The XGB and RF algorithms also performed robustly (R^2^ ≥ 0.97) with minimal calibration bias. Within the Stewart framework, performance was slightly more variable, with calibration slopes of 1.02–1.17 and lower R^2^ values (0.88–0.98). Although the Stewart–SVR model maintained good consistency (slope = 1.02, R^2^ = 0.98), the Stewart–RF model showed signs of overfitting (slope = 1.17, intercept = −1.23). The partitioned BE approach demonstrated the strongest overall alignment, combining accurate calibration with high explanatory performance. The Partitioned BE–XGBoost model achieved the optimal balance between precision and generalizability (slope = 1.00, intercept = −0.41, R^2^ = 0.99, CV-R^2^ = 0.99 ± 0.01), followed closely by Partitioned BE–SVR (R^2^ = 0.97). Collectively, the Partitioned BE models exhibited the best internal stability across folds, confirming the reproducibility of the partitioned BE approach (Table S6 and Figure 2).

#### 3.4.2. Variable Importance

**Figure 3:**
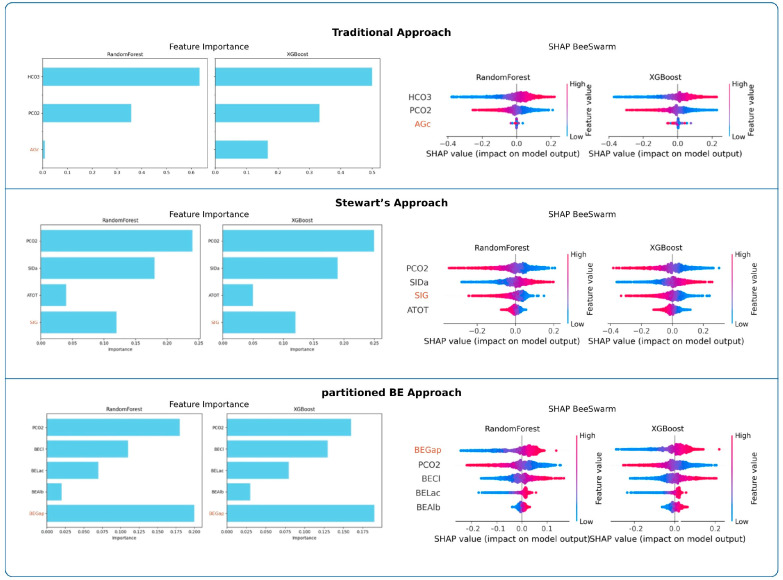
Feature importance and SHAP value analyses across frameworks. Random Forest and XGBoost importance values and SHAP beeswarm plots highlight the relative influence of predictors within each analytical framework.

Feature importance analyses are presented in Figure 3. Across both RF and XGBoost, PaCO_2_ was consistently the strongest determinant of pH in the traditional and Stewart approaches, whereas HCO_3_^−^ dominated in the traditional model. In the partitioned BE approach, BEGap emerged as the most influential predictor, surpassing other parameters. SHAP beeswarm plots confirmed these findings across models.

##### Random Forest (RF)

In the traditional approach, HCO_3_^−^ emerged as the dominant determinant of pH, whereas PaCO_2_ showed a moderate contribution and AGc had minimal influence. In the Stewart approach, PaCO_2_ and SIDa were the primary contributors, followed by SIG, while ATOT contributed only marginally. In the partitioned BE approach, BEGap, PaCO_2_, and BECl exhibited comparable importance, whereas BELac and BEAlb showed relatively minor contributions. Full numerical results are presented in Table 3.

##### Extreme Gradient Boosting (XGB)

In the traditional approach, HCO_3_^−^ emerged as the dominant determinant of pH, while PaCO_2_ had a moderate role and AGc showed minimal impact. In the Stewart model, PaCO_2_ and SIDa dominated the variable importance, followed by SIG, whereas ATOT contributed only marginally. Within the partitioned base excess model, BEGap, PaCO_2_, and BECl demonstrated comparable influence, while BELac and BEAlb remained minor contributors. Full numerical results are presented in Table 3.

##### Support Vector Regression (SVR)

In the traditional model, HCO_3_ clearly dominated as the primary determinant of pH, followed by PaCO_2_, while AGc remained negligible. In the Stewart model, PaCO_2_ and SIDa emerged as the strongest predictors, while SIG showed intermediate influence and A_TOT_ added little to the model. Within the partitioned base excess model, PaCO_2_, BEGap, and BECl demonstrated similar levels of importance, whereas BELac contributed modestly and BEAlb had minimal effect. Complete numerical values are presented in Table S4.

##### Feature Ablation Analysis

In the traditional approach (PaCO_2_ + HCO_3_^−^ + AGc), model performance was R^2^ = 0.966 (RF) and 0.972 (XGBoost). Removing AGc did not alter performance (RF: 0.966; XGBoost: 0.964), with minimal changes (ΔR^2^ = 0.000 and 0.008, respectively). Stewart Approach. In the Stewart approach (PaCO_2_ + SIDa + ATOT + SIG), model performance was R^2^ = 0.876 (RF) and 0.967 (XGBoost). Removing SIG reduced performance to 0.548 (RF) and 0.496 (XGBoost), corresponding to ΔR^2^ = 0.328 and 0.471, respectively. Partitioned BE Approach In the partitioned BE approach (PaCO_2_ + BECl + BEAlb + BELac + BEGap), model performance was R^2^ = 0.922 (RF) and 0.975 (XGBoost). Removing BEGap reduced performance to 0.588 (RF) and 0.536 (XGBoost), yielding ΔR^2^ = 0.334 and 0.439, respectively. Results are presented in Table 4.

##### External Validation

On the independent validation cohort of 1,202 ICU patients, all three frameworks demonstrated high model fit (R^2^ range: 0.85–0.98) with calibration slopes approximating unity (0.98–1.07) and small intercepts within ±0.5, indicating minimal bias (Table S7).

Among all algorithms, the Partitioned BE–XGBoost model achieved the highest external R^2^ (0.982) and the lowest error metrics (RMSE = 0.0168, MAE = 0.0079), confirming its excellent generalizability. The Partitioned BE–RF and Traditional–RF models also maintained strong alignment (R^2^ ≥ 0.97). The Stewart’s approach models yielded slightly lower R^2^ values (0.85–0.92), especially for the MLR model. Despite this, calibration slopes close to 1.0 across all models confirmed good agreement between predicted and observed pH. Overall, the Partitioned BE approach provided the best trade-off between calibration, precision, and external robustness, supporting its potential as a physiologically interpretable and transferable model for bedside acid–base assessment (Table S7).

##### Correlation and Agreement Analyses Between AGc, SIG, and BEGap

Correlation and agreement analyses between AGc, SIG, and BEGap revealed that numerical correlations were strong, while categorical agreement remained weak. Pearson correlation coefficients demonstrated a strong negative relationship between AGc and BEGap (r = −0.88) and between BEGap and SIG (r = −0.85), with a moderate-to-strong positive correlation between AGc and SIG (r = 0.72). However, Cohen’s kappa coefficients indicated only poor-to-fair agreement: AGc vs. BEGap (κ = 0.24), AGc vs. SIG (κ = 0.10), and BEGap vs. SIG (κ = 0.40).

The normal reference values for AGc, SIG, and BEGap were considered to be 7–17, 0, and 0, respectively [8,13,15,16,23]. Cross-tabulation analyses demonstrated frequent diagnostic discordance between the classical and physicochemical frameworks. Among patients with a normal AGc (7–17 mmol/L), 1228 (BEGap) and 1241 (SIG) were reclassified as unmeasured-anion (UA)-acidosis, while 62 (BEGap) and 51 (SIG) were identified as unmeasured-cation (UC)-alkalosis. Cross-tabulations are provided in SDM Tables S8–S10, presenting classifications into UA-acidosis, UC-alkalosis, or normal status.

### 3.5. Analytical Comparison Across Illustrative Cases

As shown in Table 5, the three illustrative cases exhibited complex and heterogeneous acid–base disturbances, which were most clearly and consistently characterized by the partitioned BE approach across differing clinical contexts.

## 4. Discussion

### 4.1. Findings and Comparison with Current Literature

In this study, three analytical approaches for characterizing UIs were compared using both MLR and ML models. Regression analyses showed that all three approaches explained a substantial proportion of the variability in arterial pH. The partitioned BE approach demonstrated the strongest explanatory performance, followed by the traditional approach, while the Stewart’s approach showed comparatively weaker performance in MLR. A similar pattern was observed in ML models. Although all approaches achieved high performance under non-linear conditions, the Stewart’s approach generally underperformed relative to the partitioned BE and traditional approaches. An exception was observed in the SVR model, in which the Stewart’s approach performed comparatively better.

ML models were used as complementary analytical tools to benchmark the behavior of each approach rather than to develop a deployable prediction system. Because acid–base physiology involves non-linear interactions among electrolytes, albumin, lactate, and PaCO_2_, ML enabled evaluation of these relationships as well as assessment of calibration and temporal external generalizability. The consistent superiority of the partitioned BE approach across both linear and non-linear models suggests that its performance reflects a robust physiological signal rather than an artifact of linear regression [6].

BEGap emerged as the most influential determinant of pH in both RF and XGBoost models. In the SVR model, its contribution was slightly lower than that of BECl and PaCO_2_ but remained comparable. In MLR, PaCO_2_, BEGap, and BECl showed similar importance, indicating that BEGap functions as a strong independent predictor of pH. SIG consistently ranked below PaCO_2_ and SID in all ML models and in MLR. However, it retained a meaningful contribution, in line with its role as the fourth independent variable in the Stewart approach [11,13,23]. In contrast, AGc showed minimal independent contribution to pH across all models. It consistently ranked well below HCO_3_^−^ and PaCO_2_, suggesting limited value as an independent determinant of pH. Feature ablation also showed that SIG and BEGap, but not AGc, contributed materially to model performance, highlighting their value in capturing unmeasured ion effects.

BEGap showed a strong negative correlation with both AGc and SIG. In contrast, AGc and SIG were moderately to strongly positively correlated. Previous studies have reported that SIG correlates more closely with AGc than with AG [10,13]. In addition, AGc corrected for phosphate and lactate has been shown to numerically approximate SIG [10]. In contrast, evidence regarding BEGap correlations remains limited [21]. Despite these numerical correlations, agreement between the three parameters was low, as reflected by low kappa coefficients, and they frequently classified patients differently as having UA acidosis, UC alkalosis, or normal status. Consequently, a state considered normal by one approach could indicate the presence of unmeasured ions when assessed by another, introducing diagnostic uncertainty and potential therapeutic variability [23]. For example, among 977 patients classified as having a normal AGc, the partitioned BE approach identified 348 cases of UA acidosis and 626 cases of UC alkalosis. In comparison, the Stewart’s approach classified 742 patients as having UA acidosis and 235 as UC alkalosis. These discrepancies underscore the limitations of relying solely on AGc to assess UIs.

In variable importance analyses, PaCO_2_ was the strongest determinant of pH in both modern approaches, as expected, underscoring the major impact of the respiratory component [23]. In the traditional approach, however, PaCO_2_ ranked second to HCO_3_^−^. The prominence of HCO_3_^−^ likely reflects its derivation from pH and PaCO_2_ [23]. By contrast, the prominence of SID (primarily Na^+^–Cl^−^) in the Stewart’s approach and BECl in the partitioned BE approach highlights the critical role of electrolytes in acid–base balance, consistent with previous reports [32,33,34]. In a recent study involving 438 arterial blood gas samples obtained from 71 pediatric ICU patients, artificial neural networks (ANN) were used to determine which variables most strongly influence arterial acid–base and gas exchange parameters. The ANN results showed that pH was most sensitive to changes in Cl^−^, pCO_2_, Na^+^, and lactate, in that order. However, it should be noted that UIs were not included in the analysis in that study [35].

Lactate is a key parameter in blood gas analysis and has been widely associated with morbidity and mortality [36]. However, BEGap showed a stronger association with pH than BELac in both ML models and multiple linear regression, indicating that ions beyond lactate play an important role in acid–base balance. In both the Stewart (A_TOT_) and partitioned BE (BEAlb) approaches, albumin had the smallest impact on pH. Consistent with these findings, a recent study ranked the relative effects as SIG > lactate > albumin [23].

XGBoost and RF showed the best explanatory performance, with MLR close behind. SVR produced high R^2^ in some analyses but was less consistent overall. XGBoost’s boosting framework, by capturing non-linear relationships and variable interactions, provided more stable accuracy than RF [37]. In a recent retrospective analysis of 21,541 blood gases categorized according to acid–base status, XGBoost achieved the highest accuracy at 99.66%, underscoring its value in clinical data interpretation [27]. In a retrospective single-center ICU cohort employing supervised machine learning to distinguish arterial from non-arterial blood gas samples, 150 of 33,800 samples (0.44%) were found to be mislabeled. The best-performing model was XGBoost using nine features, outperforming logistic regression in the holdout analysis [29].

Minor demographic and outcome differences were observed between the internal and external cohorts. Such variability is expected in real-world ICU populations and does not undermine external validity; rather, it supports temporal validation by testing performance under non-identical but clinically comparable conditions.

### 4.2. Clinical Implications

In a cohort of 149 critically ill patients, it was reported that the traditional approach failed to detect metabolic acidosis in 13 patients (9%), whereas these disturbances were identified using the partitioned BE approach. In addition, partitioned BE enabled a more precise quantification of the individual contributors to the acid–base disturbance [38]. Analysis of the illustrative cases highlights the limitations of traditional acid–base interpretation and the practical advantages of the partitioned BE approach. In Case 1 (urosepsis with acute kidney injury), acidemia with low bicarbonate indicated metabolic acidosis, while a normal AGc suggested isolated hyperchloremic acidosis under the traditional approach. Stewart’s analysis identified a reduced SIDa, hypoalbuminemia, and a mildly positive SIG, indicating the presence of UAs. Partitioned BE, however, provided a clear decomposition, demonstrating hyperchloremic acidosis, hypoalbuminemic alkalosis, and a smaller but distinct UA acidosis, and quantitatively showed that chloride contributed more to acidemia than UIs. In Case 2 (COPD with abdominal sepsis), a near-normal pH masked a mixed disorder. Traditional interpretation emphasized metabolic alkalosis and respiratory acidosis, whereas Stewart’s analysis suggested hypochloremia and UAs. Partitioned BE revealed coexisting hypochloremic and hypoalbuminemic alkalosis together with lactic and UA acidosis, uncovering clinically relevant acidifying processes not apparent with conventional assessment. In Case 3 (diabetic ketoacidosis), despite a pH within the physiological normal range, reduced bicarbonate and low PaCO_2_ reflected complex metabolic acidosis with respiratory alkalosis. Both Stewart and partitioned BE approaches identified hypochloremic alkalosis and marked UA accumulation. However, partitioned BE achieved this interpretation using simpler and more readily applicable parameters. Overall, these cases demonstrate that clinically relevant and complex acid–base disturbances may exist even when arterial pH remains within the physiological normal range (7.35–7.45). In routine practice, traditional approaches often focus on abnormalities only when pH falls outside this range, potentially overlooking mixed or counterbalancing processes. In contrast, the partitioned BE approach provides a transparent, quantitative, and bedside-friendly framework for identifying individual acidifying and alkalinizing components without the computational complexity of the Stewart’s approach. Thus, this approach may facilitate more rapid diagnostic clarification and treatment decisions.

### 4.3. Limitations

The main limitation of this study is its single-center, retrospective design. Although this was partly mitigated by the inclusion of a large cohort with diverse diagnostic categories and by temporal external validation, prospective multicenter studies are required to confirm generalizability. In addition, the analysis was restricted to single time-point blood gas measurements obtained at ICU admission and therefore does not capture the dynamic evolution of acid–base disturbances over time. Another limitation relates to laboratory characteristics. As all measurements were performed within a single institutional laboratory, center-specific calibration and analytical practices may limit external generalizability, despite standardized measurement protocols. Several acid–base variables used in this study (e.g., HCO_3_^−^ and SBE) are mathematically or physiologically coupled to pH and PaCO_2_ by definition. This coupling reflects an inherent property of acid–base chemistry rather than a limitation unique to our analytical approach and should be considered when interpreting variable-importance rankings, particularly within the traditional approach. This study also used complete-case analysis, which may introduce selection bias by excluding patients with missing albumin or electrolyte measurements. Although this may affect generalizability, the analyzed cohorts remained large (2274 patients in the 2018–2022 cohort and 1202 patients in the 2023–2025 cohort) and included a broad spectrum of ICU diagnoses. Nevertheless, selection bias cannot be fully excluded, and future studies using prospective data collection or imputation strategies are warranted. Finally, the present study focused on physiological and analytical associations with arterial pH and did not include outcome-based validation such as mortality, organ failure progression, or intervention thresholds. Accordingly, the findings should be interpreted as explanatory rather than prognostic, and future studies are needed to evaluate the clinical outcome implications of BEGap-guided interpretation.

## 5. Conclusions

In conclusion, this study compared the independent contributions of AGc, SIG, and BEGap to pH. While AGc showed only a limited effect, SIG and particularly BEGap emerged as strong independent determinants. Owing to its ease of calculation and the strong explanatory performance confirmed across regression and ML models, BEGap appears to be a preferable parameter for bedside assessment of unmeasured ions.

## Figures and Tables

**Table 1:** Demographic data and blood gas parameters of the patients.

Parameters	2018–2022	2023–2025
***n***	2274	1202
**Age, years**	68 (57–79)	62 (48–72)
**Male, *n* (%)**	1383 (61%)	515 (42%)
**BMI, (kg/m^2^)**	26.4 (23.8–29.8)	26 (24–29)
**APACHE II**	26 (22–31)	24 (20–28)
**SOFA score**	7 (5–10)	7 (5–9)
**Mortality, *n* (%)**	908 (40%)	420 (35%)
**pH**	7.37 (7.3–7.42)	7.37 (7.3–7.42)
**PaCO_2_ (mmHg)**	40.9 (35.0–47.7)	39.6 (34.3–45.8)
**HCO_3_^−^ (mmol/L)**	22.9 (19.8–25.3)	22.6 (19.8–24.7)
**AGc (mmol/L)**	17.6 (15.2–21.0)	18.4 (15.7–21.7)
**Na (mmol/L)**	138 (135–141)	138 (135–141)
**K (mmol/L)**	4.09 (3.63–4.59)	4.1 (3.8–4.6)
**Ca (mmol/L)**	1.17 (1.11–1.23)	1.15 (1.1–1.25)
**Mg (mmol/L)**	0.80 (0.72–0.90)	0.81 (0.74–0.92)
**Cl (mmol/L)**	104 (100–107)	103 (100–107)
**Alb (g/L)**	31.2 (26–36.4)	32.9 (28.1–38)
**[Alb]^−^ (mmol/L)**	8.5 (7–9.9)	9 (7.7–10.4)
**Lactate (mmol/L)**	1.80 (1.20–2.95)	1.7 (1.2–3)
**PO_4_ (mg/dL)**	3.8 (3.1–4.9)	3.9 (3.1–5.1)
**Pi (mmol/L)**	1.23 (1.00–1.58)	1.26 (1.02–1.64)
**[Pi]^−^ (mmol/L)**	2.2 (1.8–2.8)	2.29 (1.85–2.96)
**SBE (mmol/L)**	−1.60 (−5.30–1.40)	−2.1 (−5.4–0.5)
**BECl (mmol/L)**	−1.00 (−4.00–2.00)	0 (−3–2)
**BELac (mmol/L)**	−0.80 (−1.95–−0.20)	−0.7 (−2.2–−0.2)
**BEAlb (mmol/L)**	2.70 (1.41–4.00)	2.27 (1–3.47)
**BEGap (mmol/L)**	−1.85 (−4.96–0.55)	−2.5 (−5.7–−0.1)
**SIDa (mmol/L)**	37.7 (34.7–40.6)	38.3 (35.8–41.3)
**ATOT (mmol/L)**	10.9 (9.49–12.35)	11.6 (10.23–12.95)
**SIG (mmol/L)**	3.64 (1.42–6.35)	4.46 (2.09–7.2)

Values are presented as *n* (%) or median (IQR), as appropriate. AGc, albumin-corrected anion gap; Alb, albumin; APACHE II, Acute Physiology and Chronic Health Evaluation II; ATOT, total weak acids; BEAlb, base excess of albumin; BECl, base excess of chloride; BEGap, base excess gap; BELac, base excess of lactate; BMI, body mass index; Ca, calcium; Cl, chloride; HCO_3_^−^, bicarbonate; K, potassium; Na, sodium; PaCO_2_, arterial carbon dioxide tension; Pi, phosphate; SBE, standard base excess; SIDa, apparent strong ion difference; SIG, strong ion gap; SOFA, Sequential Organ Failure Assessment.

**Table 2:** Multiple linear regression analyses of three acid–base approaches for predicting pH.

Approach	Variable	Coefficient	95% CI	*p*	P.I.
**Traditional**	*Adjusted R^2^: 0.929; Durbin-Watson: 2.11; ANOVA: p < 0.001*				
	**HCO_3_**	0.0176	(0.0173, 0.0179)	<0.001	0.524
	**PaCO_2_**	−0.0057	(−0.0058, −0.0056)	<0.001	0.436
	**AGc**	−0.0014	(−0.0017, −0.001)	<0.001	0.039
**Stewart**	*Adjusted R^2^: 0.926; Durbin-Watson: 2.02; ANOVA: p < 0.001*				
	**PaCO_2_**	−0.0089	(−0.009, −0.009)	<0.001	0.348
	**SIDa**	0.0186	(0.018, 0.019)	<0.001	0.290
	**SIG**	−0.0193	(−0.020, −0.019)	<0.001	0.238
	**ATOT**	−0.0197	(−0.020, −0.019)	<0.001	0.124
**Partitioned BE**	*Adjusted R^2^: 0.949; Durbin-Watson: 1.89; ANOVA: p < 0.001*				
	**PaCO_2_**	−0.0058	(−0.006, −0.006)	<0.001	0.264
	**BEGap**	0.0153	(0.015, 0.016)	<0.001	0.253
	**BECl**	0.0145	(0.014, 0.015)	<0.001	0.250
	**BELac**	0.0157	(0.015, 0.016)	<0.001	0.136
	**BEAlb**	0.0153	(0.015, 0.016)	<0.001	0.097

AGc, albumin-corrected anion gap; ATOT, total weak acids; BEAlb, base excess of albumin; BECl, base excess of chloride; BEGap, base excess gap; BELac, base excess of lactate; CI, confidence interval; PaCO_2_, arterial carbon dioxide tension; P.I., predictor importance; SIDa, apparent strong ion difference; SIG, strong ion gap.

**Table 3:** Performance of machine learning models and variable importance measures across three acid–base approaches.

Approach	Algorithm	Variable	TreeImp	SHAP	Combined
**Traditional**	**RF**		*R^2^: 0.979; RMSE: 0.015; MAE: 0.009*		
		**HCO_3_**	0.632	0.066	0.349
		**PaCO_2_**	0.358	0.048	0.203
		**AGc**	0.008	0.00075	0.004
	**XGBoost**		*R^2^: 0.974; RMSE: 0.017; MAE: 0.01*		
		**HCO_3_**	0.658	0.067	0.362
		**PaCO_2_**	0.334	0.049	0.191
		**AGc**	0.007	0.002	0.004
**Stewart**	**RF**		*R^2^: 0.876; RMSE: 0.038; MAE: 0.023*		
		**PaCO_2_**	0.424	0.055	0.240
		**SIDa**	0.313	0.050	0.182
		**SIG**	0.201	0.035	0.119
		**ATOT**	0.061	0.015	0.038
	**XGBoost**		*R^2^: 0.967; RMSE: 0.019; MAE: 0.011*		
		**PaCO_2_**	0.408	0.069	0.239
		**SIDa**	0.320	0.065	0.193
		**SIG**	0.192	0.050	0.121
		**ATOT**	0.079	0.029	0.054
**Partitioned BE**	**RF**		*R^2^: 0.922; RMSE: 0.03; MAE: 0.019*		
		**BEGap**	0.349	0.045	0.197
		**PaCO_2_**	0.308	0.044	0.177
		**BECl**	0.189	0.038	0.114
		**BELac**	0.117	0.019	0.06
		**BEAlb**	0.034	0.009	0.021
	**XGBoost**		*R^2^: 0.975; RMSE: 0.017; MAE: 0.011*		
		**BEGap**	0.308	0.049	0.179
		**PaCO_2_**	0.277	0.049	0.163
		**BECl**	0.217	0.047	0.132
		**BELac**	0.149	0.024	0.086
		**BEAlb**	0.048	0.018	0.033

AGc, albumin-corrected anion gap; ATOT, total weak acids; BEAlb, base excess of albumin; BECl, base excess of chloride; BEGap, base excess gap; BELac, base excess of lactate; MAE, mean absolute error; PaCO_2_, arterial carbon dioxide tension; RMSE, root mean square error; SIDa, apparent strong ion difference; SIG, strong ion gap; SHAP, Shapley additive explanations; TreeImp, tree-based importance; Combined, combined feature importance.

**Table 4:** Feature ablation analysis across acid–base approaches. Full R^2^ reflects model performance using the complete feature set, whereas Ablated R^2^ indicates performance after removal of a framework-specific metabolic variable. ΔR^2^ denotes the performance reduction (Full R^2^–Ablated R^2^).

Approach	Model	Full R^2^	Ablated R^2^	ΔR^2^	Removed Variable
**Traditional**	**RF**	0.966	0.966	0.000	AGc
	**XGB**	0.972	0.964	0.008	AGc
**Stewart**	**RF**	0.876	0.548	0.328	SIG
	**XGB**	0.967	0.496	0.471	SIG
**Partitioned BE**	**RF**	0.922	0.588	0.334	BEGap
	**XGB**	0.975	0.536	0.439	BEGap

AGc, anion gap corrected for albumin; BEGap, base excess gap; RF, random forest; R^2^, coefficient of determination; SIG, strong ion gap; XGB, extreme gradient boosting.

**Table 5:** Summary of clinical context and analytical acid–base parameters across three illustrative cases.

Parameter	Case 1	Case 2	Case 3
**Clinical Context**	69M, urosepsis + AKI	72M, COPD + abdominal sepsis	32F, abdominal pain, DKA/AKI
**pH**	7.32	7.37	7.45
**PaCO_2_ (mmHg)**	29.1	55	18
**HCO_3_^−^ (mmol/L)**	16	31	18
**SBE** **(mmol/L)**	−10.1	+5.2	−9
**Na^+^ (mmol/L)**	145	136	125
**K^+^ (mmol/L)**	2.3	3.3	2.4
**Cl^−^ (mmol/L)**	121	92	86
**Ca^2+^ (mmol/L)**	1.43	1.2	1.1
**Mg^2+^ (mmol/L)**	0.93	0.91	0.94
**Pi (mmol/L)**	1.6	1.4	1.2
**Lactate (mmol/L)**	1	3.4	2.0
**Albumin (g/L)**	27	24	38
**AGc (mEq/L)**	14	20.8	24.4
**SIDa (mEq/L)**	27.7	46	41.4
**A_TOT_ (mmol/L)**	10.1	13	13
**SIG (mEq/L)**	+1.6	+2	+10.4
**BECl (mEq/L)**	−11	+9	+7
**BEAlb (mEq/L)**	+3.75	+4.5	+1
**BELac (mEq/L)**	0	−2.4	−1
**BEGap (mEq/L)**	−2.85	−6.9	−16

AGc, albumin-corrected anion gap; ATOT, total weak acids; AKI, acute kidney injury; BEAlb, albumin effect on base excess; BECl, chloride effect on base excess; BEGap, base excess gap; BELac, lactate effect on base excess; Ca^2+^, ionized calcium; Cl^−^, chloride; DKA, diabetic ketoacidosis; F, female; HCO_3_^−^, bicarbonate; K^+^, potassium; Lactate, lactate; M, male; Mg^2+^, magnesium; Na^+^, sodium; PaCO_2_, partial pressure of carbon dioxide; Pi, inorganic phosphate; SBE, standard base excess; SIDa, apparent strong ion difference; SIG, strong ion gap.

## Data Availability

The data presented in this study are available on request from the corresponding author (the data are not publicly available due to ethical restrictions of the hospital).

## Supplementary Materials

The following supporting information can be downloaded at: https://www.mdpi.com/article/10.3390/diagnostics16030427/s1, Table S1. PROBAST-AI Checklist for Risk of Bias and Applicability Assessment in the Present Study; Table S2. Hyperparameter settings used for machine learning models; Table S3. Distribution of admission diagnoses in the internal and external cohorts; Table S4. Permutation importance (PI) results for Support Vector Regression (SVR) models across the traditional, Stewart, and partitioned base excess frameworks, including corresponding model fit metrics (R^2^, RMSE, MAE); Table S5. Cross-validation results of machine learning models for pH prediction; Table S6. Internal calibration and discrimination metrics across analytical frameworks and algorithms; Table S7. External validation performance of all modeling frameworks on the independent test cohort (n = 1202); Table S8. Cross-tabulation: AGc vs. BEGap; Table S9. Cross-tabulation: AGc vs. SIG; Table S10. Cross-tabulation: BEGap vs. SIG.
