# Supplementary material for: Unveiling the Gaps: Machine Learning Models for Unmeasured Ions
Source: Diagnostics (Basel). 2026 Feb 1;16(3):427. doi: 10.3390/diagnostics16030427 (PMC12897467; doi:10.3390/diagnostics16030427)
Supplement: Supplementary file 1 [file diagnostics-16-00427-s001.zip › diagnostics-4076826-supplementary.pdf]

Supplementary Digital Material

- **Table S1.** PROBAST-AI checklist for risk of bias and applicability.

- **Table S2.** Hyperparameter settings used for machine learning models

- **Table S3.** Admission diagnoses comparison (internal vs. external cohort).

- **Table S4.** Permutation importance (PI) results for Support Vector Regression (SVR) models across the traditional, Stewart, and partitioned base excess frameworks, including corresponding model fit metrics (R^2, RMSE, MAE).

- **Table S5.** Cross-validation results (internal performance).

- **Table S6.** Internal calibration and discrimination metrics.

- **Table S7.** External validation metrics (independent cohort).

- **Tables S8, S9 and S10.** Cross-tabulations between AGc, SIG, and BEGap

Summary of the Prediction model Risk Of Bias ASsessment Tool–Artificial Intelligence (PROBAST-AI) applied to evaluate potential sources of bias, data leakage, and model applicability across development and validation phases (**Table S1**).

Table S1. PROBAST-AI Checklist for Risk of Bias and Applicability Assessment in the Present Study

Term	Definition	How addressed in this study
**Algorithmic bias**	When model predictions disadvantage groups unjustifiably.	Not applicable; no subgroup stratification (ICU data, same sampling).
**Apparent performance**	Model performance on training data.	Reported as internal CV-R^2 and RMSE (Table 3S).
**External validation**	Evaluation using unseen data.	Performed on 2023–2025 ICU cohort (n=1202).
**Calibration**	Agreement between observed and predicted outcomes.	Assessed using calibration plots (Figure 2) and slopes/intercepts (Table 3S).
**Data leakage**	Overlap between training and test datasets.	Prevented via temporal split (2018–2022 vs 2023–2025).
**Discrimination**	Ability to separate outcomes.	Not applicable (continuous regression task, not binary).
**Evaluation or test data**	Data used to estimate performance.	External dataset (2023–2025).
**Internal validation**	Performance assessment using resampling on training data.	10-fold CV performed (internal CV-R^2 reported).
**Machine learning**	Algorithms that learn patterns from data.	RF, XGB, SVR used.
**Model evaluation**	Measuring performance metrics.	R^2, RMSE, MAE, slope, intercept used.
**Outcome**	The variable predicted by the model.	Arterial pH (continuous outcome).
**Predictor**	Variables used as model inputs.	Na^+, Cl^-, pCO_2, lactate, albumin, BE components.
**Validation data**	Data reserved for final model testing.	External cohort (2023–2025).

Model hyperparameters and preprocessing steps are reported in detail in the **Table S2.**

Table S2. Hyperparameter settings used for machine learning models

Model	Hyperparameters (as implemented)
Random Forest	n_estimators=600; max_depth=None (default); min_samples_split=2 (default); min_samples_leaf=1 (default); random_state=42; n_jobs=−1
XGBoost	n_estimators=800; learning_rate=0.05; max_depth=6; subsample=0.9; colsample_bytree=0.9; reg_lambda=1.0; tree_method=hist; random_state=42; n_jobs=−1
SVR (RBF)	kernel=rbf; C=10.0; epsilon=0.01; gamma=scale

Comparison of admission diagnoses between the internal development cohort (2018–2022, n = 2,274) and the external validation cohort (2023–2025, n = 1,202). The proportion of respiratory and circulatory disorders remained highest in both cohorts, whereas renal, metabolic, and postoperative cases were relatively more frequent in the external group (**Table S3**).

Table S3. Distribution of admission diagnoses in the internal and external cohorts

Diagnosis	Internal cohort (2018–2022, n = 2274)	External cohort (2023–2025, n = 1202)
**Renal disorders**	74 (3.3 %)	63 (5.2 %)
**Sepsis**	304 (13.4 %)	129 (10.7 %)
**Metabolic disorders**	59 (2.6 %)	49 (4.1 %)
**Circulatory disorders**	549 (24.1 %)	**288 (23.9 %)**
**Trauma**	181 (8.0 %)	88 (7.3 %)
**Intoxication**	57 (2.5 %)	31 (2.6 %)
**Gastrointestinal disorders**	72 (3.2 %)	42 (3.5 %)
**Postoperative cases**	147 (6.5 %)	98 (8.1 %)
**Respiratory disorders**	**558 (24.5 %)**	214 (17.8 %)
**Neurological conditions**	96 (4.2 %)	54 (4.5 %)
**Malignancy**	131 (5.8 %)	75 (6.2 %)
**Hematologic disorders**	46 (2.0 %)	25 (2.1 %)

Complete numerical values for Support Vector Regression (SVR) are presented in **Table S4.**

Table S4. Permutation importance (PI) results for Support Vector Regression (SVR) models across the traditional, Stewart, and partitioned base excess frameworks, including corresponding model fit metrics (R^2, RMSE, MAE).

Algorhytm	Variable	PI
**Traditional** *R^2: 0.934; RMSE:0.028; MAE:0.019*		
	**HCO_3**	1.126
	**$PaCO_2$**	0.836
	**AGc**	0.053
**Stewart** *R^2: 0.996; RMSE:0.006; MAE:0.005*		
	**$PaCO_2$**	2.040
	**SIDa**	1.904
	**SIG**	1.186
	**ATOT**	0.411
**Partitioned BE** *R^2: 0.989; RMSE:0.011; MAE:0.005*		
	**BECl**	0.885
	**$PaCO_2$**	0.845
	**BEGap**	0.840
	**BELac**	0.334
	**BEAlb**	0.129

AGc, albumin-corrected anion gap; ATOT, total concentration of non-volatile weak acids; BEAlb, base excess due to albumin; BECl, base excess due to chloride; BEGap, base excess gap (unmeasured ions); BELac, base excess due to lactate; HCO_3^-, bicarbonate; MAE, mean absolute error; $PaCO_2$, arterial partial pressure of carbon dioxide; PI, permutation importance; RMSE, root mean square error; R^2, coefficient of determination; SIDa, apparent strong ion difference; SIG, strong ion gap; SVR, support vector regression.

Cross-validation results of machine learning models are summarized in **Table S5**.

Table S5. Cross-validation results of machine learning models for pH prediction

Model	R^2 (mean ± SD)	RMSE (mean ± SD)	MAE (mean ± SD)
**XGB**	0.975 ± 0.010	0.017 ± 0.003	0.009 ± 0.001
**RF**	0.971 ± 0.014	0.019 ± 0.004	0.009 ± 0.001
**Linear Regression**	0.971 ± 0.003	0.019 ± 0.002	0.011 ± 0.000
**SVR**	0.930 ± 0.037	0.029 ± 0.007	0.013 ± 0.002

MAE, mean absolute error; RF, random forest; RMSE, root mean square error; SVR, support vector regression; XGB, extreme gradient boosting.

Internal calibration and discrimination metrics across analytical frameworks are summarized in **Table S6**.

Table S6. Internal calibration and discrimination metrics across analytical frameworks and algorithms

Framework	Algorithm	Calibration Slope	Intercept	Calibration-in-the-large	MSE (Brier-like)	RMSE (test)	MAE (test)	R^2 (test)	CV-R^2 (mean ± SD)
Traditional	**MLR**	1.05	−0.35	0.00003	0.00081	0.028	0.018	0.93	0.93 ± 0.01
	**RF**	1.01	−0.11	0.00144	0.00026	0.016	0.010	0.98	0.96 ± 0.02
	**XGB**	1.03	−0.24	0.00133	0.00043	0.021	0.013	0.97	0.95 ± 0.02
	**SVR**	0.99	0.03	−0.00089	0.00017	0.013	0.008	0.99	0.96 ± 0.02
Stewart	**MLR**	1.08	−0.57	−0.00141	0.00091	0.030	0.019	0.93	0.93 ± 0.01
	**RF**	1.17	−1.23	−0.00110	0.00151	0.039	0.024	0.88	0.89 ± 0.02
	**XGB**	1.14	−1.02	−0.00008	0.00092	0.030	0.017	0.92	0.93 ± 0.02
	**SVR**	1.02	−0.13	−0.00286	0.00019	0.014	0.007	0.98	0.96 ± 0.04
Partitioned BE	**MLR**	1.05	−0.34	−0.00014	0.00059	0.024	0.015	0.95	0.95 ± 0.01
	**RF**	1.12	−0.91	0.00053	0.00095	0.031	0.020	0.92	0.90 ± 0.02
	**XGB**	**1.00**	−0.41	−0.00022	0.00043	0.019	0.010	**0.99**	**0.99 ± 0.01**
	**SVR**	1.09	−0.65	0.00031	0.00049	0.022	0.013	0.97	0.95 ± 0.01

Calibration and discrimination results of the traditional, Stewart, and partitioned base excess (BE) frameworks evaluated in an independent validation cohort. Values represent the coefficient of determination (R^2), root mean square error (RMSE), mean absolute error (MAE), calibration slope, and intercept. Calibration slopes near 1.0 and intercepts close to 0 indicate accurate model calibration. Among all models, the **Partitioned BE–XGBoost** combination achieved the highest external alignment (R^2 = 0.982, RMSE = 0.0168, MAE = 0.0079), confirming excellent generalizability. SHAP and permutation importance analyses were performed only during internal validation to prevent post-hoc bias or data leakage (**Table S6**).

External validation performance of all modeling frameworks are summarized in **Table S7.**

Table S7. External validation performance of all modeling frameworks on the independent test cohort (n = 1,202).

Framework	Algorithm	R^2 (External)	RMSE	MAE	Calibration Slope	Intercept
**Traditional**	RF	**0.971**	**0.0168**	0.0084	0.98	+0.06
Traditional	SVR	0.970	0.0172	0.0082	0.98	+0.16
Traditional	XGB	0.963	0.0190	0.0105	1.03	−0.08
Traditional	MLR	0.932	0.0252	0.0155	1.07	−0.48
**Partitioned BE**	XGB	**0.982**	**0.0168**	0.0079	0.99	+0.04
Partitioned BE	RF	0.974	0.0172	0.0081	0.98	+0.15
Partitioned BE	MLR	0.973	0.0190	0.0104	1.01	−0.06
Partitioned BE	SVR	0.934	0.0252	0.0152	1.06	−0.46
**Stewart**	SVR	**0.918**	**0.0281**	0.0164	1.05	−0.38
Stewart	XGB	0.916	0.0285	0.0126	0.99	+0.06
Stewart	RF	0.902	0.0309	0.0164	1.04	−0.32
Stewart	MLR	0.848	0.0383	0.0227	1.04	−0.32

Cross-tabulations are provided in SDM **Tables S8, S9**, and **S10**, presenting classifications into unmeasured anion (UA)-acidosis, unmeasured cation (UC)-alkalosis, or normal status.

AGc vs BEGap (see Table S8):
Cross-tabulation demonstrated frequent discrepancies between AGc and BEGap categories, with many patients classified as normal by AGc falling into UA-acidosis or UC-alkalosis according to BEGap.

AGc vs SIG (see Table S9):
Although AGc >17 generally corresponded to SIG >0, patients within the AGc normal range were often reclassified by SIG, indicating limited categorical agreement.

BEGap vs SIG (see Table S10):
Most patients with BEGap <0 were also identified as UA-acidosis by SIG, whereas BEGap >0 showed mixed distribution across both SIG >0 and SIG <0 categories.

Table S8. Cross-tabulation: AGc vs BEGap

	**BEGap = 0 Normal**	**BEGap < 0 UA-Acidosis**	**BEGap > 0 UC-Alkalosis**	**Total**
**17 ≥ AGc ≥ 7 Normal**	3	348	626	977
**AGc > 17 UA-Acidosis**	2	1228	62	1292
**AGc < 7 UC-Alkalosis**	0	0	4	4
**Total**	5	1576	692	2273

Table S9. Cross-tabulation: AGc vs SIG

	**SIG > 0 UA-Acidosis**	**SIG < 0 UC-Alkalosis**	**Total**
**17 ≥ AGc ≥ 7 Normal**	742	235	977
**AGc > 17 UA-Acidosis**	1241	51	1292
**AGc < 7 UC-Alkalosis**	0	4	4
**Total**	1983	290	2273

Table S10. Cross-tabulation: BEGap vs SIG

	**SIG > 0 UA-Acidosis**	**SIG < 0 UC-Alkalosis**	**Total**
**BEGap = 0 Normal**	5	0	5
**BEGap < 0 UA-Acidosis**	1537	39	1576
**BEGap > 0 UC-Alkalosis**	441	251	692
**Total**	1983	290	2273
